# Efficacy of Diclofenac Transdermal Patch in Therapeutic Extractions: A Literature Review

**DOI:** 10.7759/cureus.30411

**Published:** 2022-10-18

**Authors:** Akshata Awachat, Deepankar Shukla, Nitin D Bhola

**Affiliations:** 1 Department of Oral and Maxillofacial Surgery, Sharad Pawar Dental College and Hospital Sawangi, Datta Meghe Institute of Medical Sciences (DU), Wardha, IND; 2 Department Oral and Maxillofacial Surgery, Sharad Pawar Dental College and Hospital Sawangi, Datta Meghe Institute of Medical Sciences (DU), Wardha, IND

**Keywords:** rescue analgesia, efficacy, orthodontic extraction, transdermal patch, diclofenac

## Abstract

Diclofenac sodium is a nonsteroidal anti-inflammatory drug that effectively manages pain following therapeutic extractions. Post-extraction pain is commonly treated with non-steroidal anti-inflammatory drugs (NSAIDs). In addition to their high bioavailability and long duration of action, transdermal NSAIDS have several other advantages. The review tries to understand and elucidate the use of transdermal patches, here Diclofenac, as a postoperative pain management modality. Drug delivery is one of the essential aspects of drug administration where transdermal patches are to be found equally effective when compared to oral administration of drugs. Various analgesics can be administered as patches, for example, ketoprofen, diclofenac, etc. There are also comparative studies between diclofenac and ketoprofen to see and understand the efficacy of transdermal patches compared with oral administration. Compared to oral administration, transdermal patches offer numerous benefits. These include avoidance of first-pass metabolism, sustained and non-rapid absorption, steady plasma levels that remain for prolonged periods, lack of patient dependence on drugs, prevention of gastric distress, and flexibility of stopping delivery of medications by simply removing the patch. This review aims to examine the diclofenac transdermal patch's effectiveness in reducing postoperative pain after orthodontic extraction.

## Introduction and background

**Figure 1:**
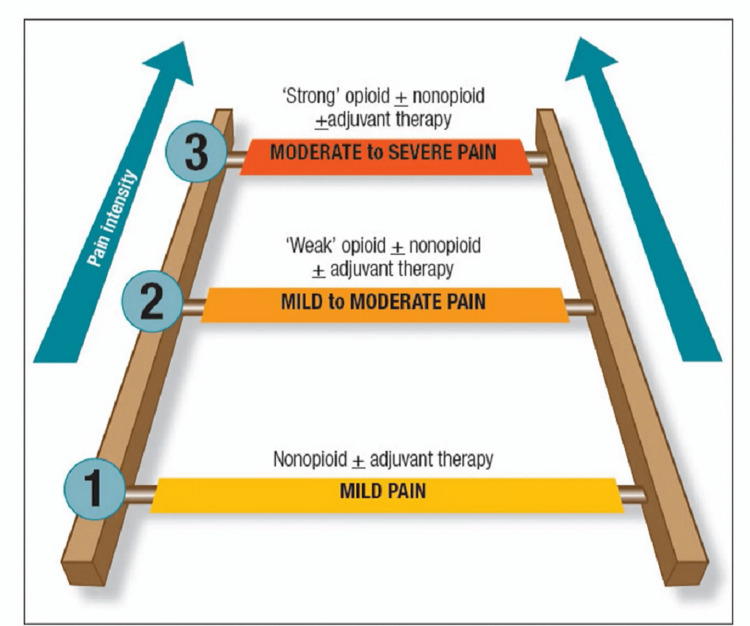
WHO analgesic pain ladder used in the management of pain Source [4].

Drug delivery is an essential aspect of drug administration, where transdermal patches are found to be equally effective compared to oral drug administration. Various analgesics can be administered as patches, e.g., ketoprofen, diclofenac, etc. There are also comparative studies between diclofenac and ketoprofen that compare their efficacy in transdermal and oral administration [1]. Several routes of administration are available, including oral, transdermal, parenteral, neuraxial, and rectal [2]. Postoperative dental pain can be treated safely with non-steroidal anti-inflammatory drugs (NSAIDs) [3]. The WHO pain ladder (Figure 1) describes pain in terms of intensity [4].

In any dental treatment, the dentist's primary goal is to relieve pain as effectively as possible for patients. The experience of pain is a complication involving specific sensations and reactions that occur in response, making it both a physiological and psychological challenge. Monitoring pain relief after surgery using the Visual Analog Scale (VAS) has become an essential postoperative quality assessment procedure because it offers considerable physiological benefits. The dentist must select an analgesic regimen to help eliminate pain effectively while minimizing side effects. Can manage pain through opioids as well as non-opioid medications. Several routes of administration are available (oral, transdermal, neuraxial, intravenous [IV], and regional). As an alternative method of achieving analgesia, transdermal patches are gaining popularity [2].

Transdermal patches, commonly referred to as transdermal delivery systems, are non-invasive systems for delivering medications into the dermis. This method of drug delivery is a potential alternative to oral ingestion of medications and can improve patient compliance. The first transdermal patch was developed in the 1970s, and the Food and Drug Administration (FDA) approved it as a motion sickness treatment in 1979 [5]. It is an alternative to oral administration and could improve acceptance by reducing adverse reactions. It also avoids the pain associated with IV and intramuscular (IM) routes. This form of nonsteroidal anti-inflammatory drugs (NSAIDs), which are applied topically, can be administered systemically in low concentrations. By delivering the medication transdermally, it is possible to reduce the strength of action and the ensuing side effects associated with oral delivery. This article compares the effectiveness of the diclofenac transdermal patch and the need for rescue analgesia within the first 24 hours [6].

**Figure 2:**
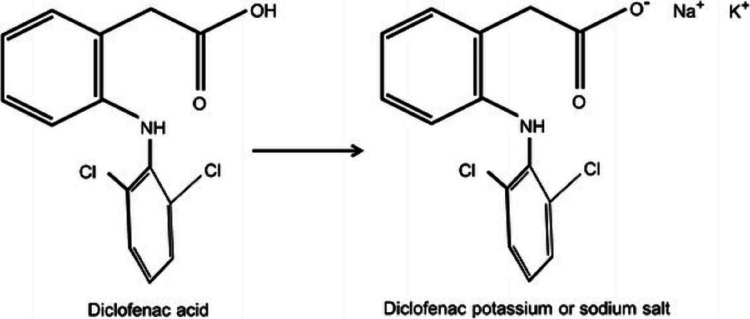
Chemical structure of diclofenac drug products Source [12].

Diclofenac is a NSAIDs that inhibits both cyclooxygenase (COX) -1 and cyclooxygenase (COX) -2 enzymes. NSAIDs inhibit the synthesis of prostanoids (prostaglandin [PG]-E2, PGD2, PGF2, prostacyclin [PGI2], and thromboxane [TX] A2) by binding to COX isozymes [7-9]. PGE2 is the dominant prostanoid produced in inflammation, and inhibiting its synthesis by NSAIDs is believed to be the primary mechanism of these agents' potent analgesic and anti-inflammatory properties (Figure 2) [9-11].

## Review

The benefits of using transdermal delivery systems

In comparison to oral administration, transdermal patches offer numerous advantages. Among them is the avoidance of first-pass metabolism, sustained and non-rapid absorption, stable plasma levels for prolonged periods, the absence of drug dependence, the prevention of gastric distress, and the ability to stop drug delivery by simply taking off the patch (Table 1) [1].

**Table 1:** Benefits of transdermal patches Source [5].

Sr. No.	Benefits of transdermal patches
1.	Transdermal patches are a non-invasive, non-painful way of administering medications.
2.	The transdermal patches deliver a variety of drugs that are absorbed in the small intestine, but not well in the stomach, and they are extensively metabolized in the liver.
3.	Drugs are delivered through transdermal patches over a long period.
4.	Transdermal patches allow abrupt termination of medication delivery.
5.	Compared to oral medications and supplements, transdermal patches have fewer side effects.
6.	Patients are comfortable using and remembering transdermal patches.
7.	In therapeutic delivery systems, a reservoir of drugs is present, and its controlled release characteristics allow the active properties of drugs with short plasma half-lives to be prolonged.
8.	Those who have cognitive disabilities or who are disabled and cannot self-medicate can use transdermal patches as an alternative.

Use of diclofenac as a transdermal patch

**Figure 3:**
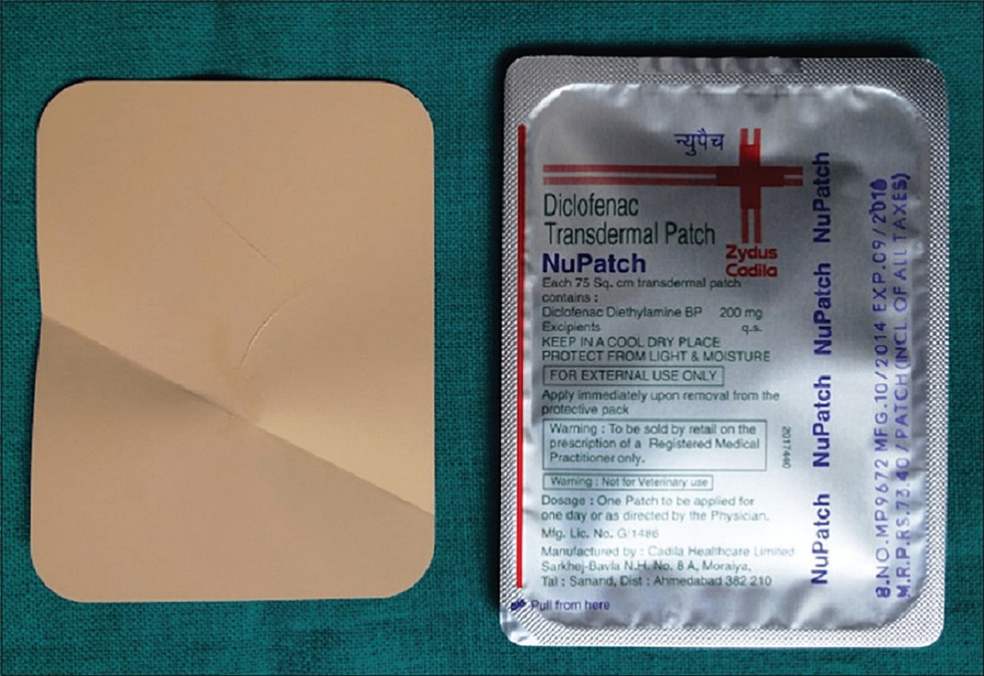
Diclofenac transdermal patch Source [16].

One of the many nonsteroidal anti-inflammatory medications used in clinical practice, diclofenac is frequently used to manage postoperative pain. According to Selvi et al., intramuscular Diclofenac and transdermal Diclofenac patches (TDP) effectively provided analgesia following third molar extractions. The study showed that a Diclofenac patch provided adequate analgesia to manage mild to moderate pain following therapeutic extractions [13]. Furthermore, Bhaskar et al. compared oral versus transdermal Diclofenac administration. They studied the satisfaction of their study patients with the transdermal patch, which produced analgesia comparable to that of the said supplement [14]. Analgesic efficacy of transdermal and intramuscular diclofenac postoperatively in orthognathic surgery patients was studied by Perepa et al. In their study, they found that diclofenac patches were a non-invasive, effective method to reduce postoperative pain without side effects. The results emphasized in this study have been reported by other studies in which patch compliance was higher than other methods of the drug (Figure 3) [15].

Medicated skin patches are adhesive patches applied cutaneously to carry a specific drug dose via the skin directly into circulation. The use of transdermal delivery is regarded as a reliable way to relieve postoperative pain due to the absence of gastrointestinal problems associated with oral administration and the risk associated with invasive intramuscular or intravenous injections. As the transdermal patch is applied to the skin, the drug is absorbed into the dermis and distributed into the capillaries for systemic absorption. Consistent and uniform serum drug levels result from constant absorption across the dermis. Pharmacokinetic data show that transdermal NSAIDs patches exert therapeutically. Effect with higher tissue concentrations and low plasma concentrations that do not adversely affect the system. Advanced therapeutic delivery systems have emerged as an essential topic in pharmaceutical technology and are one of the most recently developed pharmaceutical products in the global market [17]. Thus, diclofenac sodium is reportedly used for topical applications [18].

Drawbacks of using transdermal patch

However, despite their benefits, transdermal patches have certain drawbacks, which are shown in Table 2.

**Table 2:** Drawbacks of transdermal patch Source [19].

Sr. No.	The drawbacks of transdermal delivery systems
1	Possible allergic reactions such as rashes, itching, swelling, etc. at the site where transdermal patches are applied.
2	Drugs with greater molecular sizes (over 1000) are more difficult to absorb.
3	There is variation in the skin's barrier function based on its location.
4	Transdermal patches are costlier when compared to tablets.

Discussion

**Figure 4:**
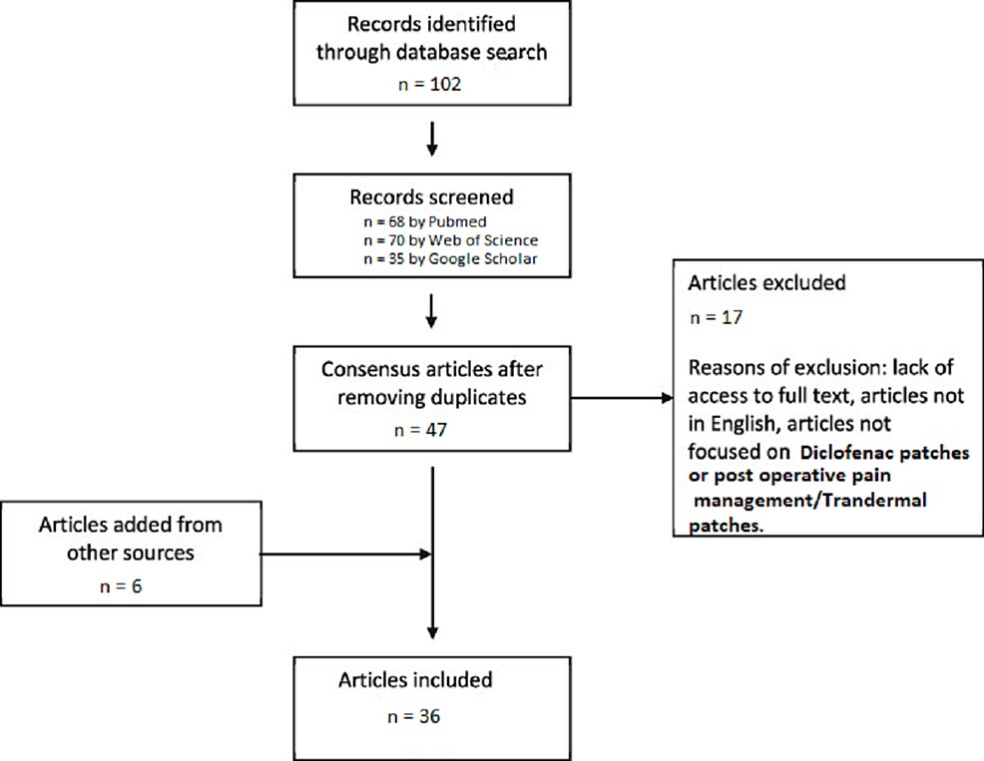
Flow diagram of literature search using PubMed, Web of Science, and Google Scholar

This review discusses the diclofenac transdermal patch's effectiveness in reducing postoperative pain after orthodontic extraction. In our report, we analyzed previously available literature discovered through a thorough search of several sources. In PubMed, Web of Science, and Google Scholar, we searched for current related data and literature to better understand the effectiveness of transdermal diclofenac patches (Figure 4).

A study conducted by Bhaskar, Kapoor, and Ragini et al. in 2013 evaluated postoperative pain within three consecutive days in 20 subjects with a pain measurement scale and a pain relief measure. Patients with transdermal patches were given paracetamol tablets as an emergency treatment. According to the results, transdermal diclofenac patch 100 mg and diclofenac tablets showed a marked reduction in pain intensity from day 1 to day 3. After three days, the transdermal patch and the oral diclofenac showed an easing of pain. According to their findings, transdermal diclofenac 100 mg was just as effective as oral diclofenac tablets, plus it was more patient-friendly [14]. Krishnan et al. in 2015 evaluated the effectiveness of diclofenac transdermally and orally in post-extraction pain management in extractions. They divided the 40 patients into six groups, each with 40 non-tender molars that could not be salvaged. An evaluation of postoperative pain was performed after six and 12 hours using the Visual Analog Scale. Patches containing diclofenac did not show a statistically significant effect in efficacy from diclofenac sodium oral tablets in managing postoperative molar extraction pain [20].

Bachalli et al. studied the effects of transdermal diclofenac patches and oral diclofenac following surgical extraction of an impacted third molar. They evaluated the postoperative pain within two hours, four hours, eight hours, 12 hours, and 24 hours on three consecutive days. On a postoperative day, oral diclofenac 100 mg was statistically and clinically more effective than a transdermal diclofenac patch. However, oral diclofenac and a diclofenac patch were similarly effective at relieving postoperative pain on days 2 and 3. A transdermal diclofenac patch can be used as an alternative to oral diclofenac during the extraction of an impacted mandibular molar. However, the analgesic effect is less in the immediate postoperative period [21]. Diclofenac may be well tolerated in renally impaired patients when used at the lowest effective dose for the shortest duration [22].

In 2015, Bhargava et al. compared postoperative pain after abdominal surgeries with diclofenac intramuscular injection and transdermal diclofenac patches. Applied transdermal diclofenac patches an hour before surgery, and 75 mg of diclofenac injections were injected 30 minutes before surgery ended. A Visual Analog Scale was used to evaluate the postoperative pain in the immediate postoperative period: four hours after surgery, eight hours after surgery, 12 hours after surgery, and 24 hours after surgery. Transdermal diclofenac groups had a mean time of 7.21 hours first supplement of analgesia, while oral diclofenac groups had a mean time of 7.43 hours first supplement of analgesia. The authors found that a diclofenac patch is as helpful in providing postoperative analgesia as a diclofenac intramuscular injection [23].

In a study by Krishna and Natraj in 2012, a 100 mg diclofenac patch and a 75 mg diclofenac injection were compared as an analgesic postoperatively for subarachnoid block-induced lower limb surgeries. Diclofenac 100 mg transdermally and 75 mg intramuscularly were administered to the study group at the beginning and the control group at the end of the surgery, respectively. The Visual Analog Scale measured postoperative pain after two hours and six hours. Those with a score greater than or equal to 5 requested rescue anesthesia. For injection of tramadol 2 mg/kg, the meantime to achieve rescue analgesia was approximately seven hours in the control group and eight hours and six minutes in the study group. A transdermal diclofenac patch provides the same relief for acute postoperative pain as an intramuscular diclofenac patch without side effects [24].

The tolerability of diclofenac transdermal patches in the treatment of sports injuries was evaluated by a randomized, placebo-controlled, double-blind study conducted by Predel et al. in 2003. The study involved 120 patients. Patients were assessed twice a day for seven days for their tenderness to pressure. It has been shown that patients treated with the diclofenac patch displayed a clinically significant and statistically significant reduction in pain scores and were able to stop experiencing pain sooner than the placebo group, as well as a more excellent tenderness ratio concerning the contralateral limb than an injured limb. It was practical to use the patch of diclofenac; acute traumatic blunt and soft tissue injuries treated with this medication reported no significant adverse events [25]. In a meta-analysis of 22 using a database search on double-blinded placebo-controlled trials on the topic of NSAIDs for acute pain, Mason et al. concluded that transdermal diclofenac patches are used to treat osteoarthritis and sports-related injuries [26].

A double-blind study was conducted with 136 patients; Bailey et al. investigated the effectiveness of using diclofenac 50 mg and aspirin 600 mg for pain after removing the third molar. Diclofenac dispersible tablets reduced the intensity of pain in patients. Aspirin and diclofenac dispersible tablets reduced post-extraction pain equally, but diclofenac was more effective at opening the mouth during extraction of impacted third molars than soluble aspirin [27].

In 2004, Joshi et al., diclofenac sodium, ibuprofen, and paracetamol with codeine were tested before surgery. One hundred nineteen patients scheduled for surgery on a daycare basis to remove their third molar under general anesthesia were randomly assigned to a double-blinded, randomized study. Use a VAS (visual analogue scale) and a VAS (verbal rating scale) to evaluate postoperative pain within 15 minutes, 30 minutes, one hour, three hours, six hours, and 24 hours. Analgesics were required for an additional 17 minutes for the placebo group and 32 minutes for the diclofenac group after surgery. In conclusion, paracetamol, Ibuprofen, diclofenac 600 mg, and codeine 60 mg were equally effective in managing postoperative discomfort when teeth were removed [28].

Devireddy et al. reported that orally administered diclofenac could cause complications in the gastrointestinal system (GI). The diclofenac patch was applied transdermally. Diclofenac sodium was administered orally at the control sites. TDP effectively reduced postoperative pain after root coverage with subepithelial connective tissue grafts. In contrast to oral administration, TDP did not cause GI complications, resulting in a higher level of pain tolerance [29].

According to Sanjay Talnia et al., it was shown that for consecutive postoperative days, transdermal diclofenac patches were slightly more effective at controlling postoperative pain in orthodontic extractions than oral diclofenac tablets. Still, no significant difference was observed using the Chi-square test (P > 0.05) among their study population. Premolar orthodontic extraction pain was successfully managed with a transdermal diclofenac patch, with fewer systemic side effects. Despite this, transdermal patches may be limited by their cost and availability [30].

It is noteworthy that, according to the study conducted by Chaitanya et al., diclofenac transdermal patches have shown significant improvements in moderate pain. Compared to the transdermal route, diclofenac administered orally and intravenously significantly reduced pain scores and showed transdermal patches to be effective in mild pain cases with better patient compliance and well-tolerated by patients [31].

Raja Rajeswari et al. reviewed postoperative pain and used a numerical rating scale, a verbal rating scale, and a pain relief scale to measure postoperative pain. All patients had comparable demographics and intraoperative and postoperative characteristics. A Mann-Whitney test was used to analyze the data obtained from the three subjective scales. Oral and transdermal delivery of diclofenac had no statistically significant differences in scores. Three patients reported gastric irritation and a mild burning sensation when taking oral diclofenac, while none developed any adverse effects while using the transdermal patch. Transdermal diclofenac patches have a superior safety profile and better gastric tolerability compared with oral diclofenac patches, despite having the same efficacy [32,33].

In a study conducted by Samal et al., transdermal diclofenac was found to be a promising analgesic modality to manage mild to moderate postoperative pain following impacted third molar extractions. Diclofenac transdermal is an advantageous alternative to oral and parenteral routes of drug administration because of its proven analgesic efficacy, patient compliance, and side-effect profile. Therefore, it can be used for post-surgical and post-traumatic pain. More extensive clinical trials are needed to establish the scope of transdermal diclofenac in other oral surgical procedures [34].

Khan et al. found that ketoprofen and diclofenac transdermal patches provided adequate postoperative analgesia, but the ketoprofen group needed fewer rescue analgesics [32]. According to Funk et al., diclofenac patches provide significantly better pain relief following arthroscopic shoulder surgery than tablets [35]. Similarly, Karabayirli et al. concluded that transdermal diclofenac reduced postoperative pain as effectively as intramuscular diclofenac [36].

## Conclusions

The analgesic efficacy of diclofenac transdermal patches is promising. Pain in dentistry is inevitable and can often plague both the patient and the dentist. Future developments may make it easier to administer analgesics transdermally. Due to improved delivery and a more comprehensive selection of painkillers, this method is expected to become more popular and widely used. Compared with oral treatment, the transdermal route is the most successful innovative research area in the new drug delivery system. A transdermal drug delivery system (TDDS) is the most efficient, safest, and easiest way for systemic drug delivery. Longer clinical trials with a larger sample are required to determine the true scope of the transdermal diclofenac patch.
